# A Personalized Data Dashboard to Improve Compliance with Ecological Momentary Assessments in College Students: Protocol for a Microrandomized Trial

**DOI:** 10.2196/57664

**Published:** 2024-11-11

**Authors:** Stephanie T Lanza, Ashley N Linden-Carmichael, Danny Wang, Sandesh Bhandari, Samuel W Stull

**Affiliations:** 1 Department of Biobehavioral Health Pennsylvania State University University Park, PA United States; 2 Edna Bennett Pierce Prevention Research Center Pennsylvania State University University Park, PA United States

**Keywords:** ecological momentary assessments, data dashboard, study compliance, microrandomized trial, intensive longitudinal data, EMA, adolescents, substance use, wearables

## Abstract

**Background:**

Ecological momentary assessments (EMA) are ideal for capturing the dynamic nature of young adult substance use behavior in daily life and identifying contextual risk factors that signal higher-risk episodes. These methods could provide a signal to trigger real-time intervention delivery. Study compliance and engagement are common barriers to participation but may be improved by personalizing messages. This study compares compliance outcomes between one group of young adults receiving standard (generic) prompts at each assessment and another group that received additional personalization and an updated data dashboard (DD) showing study progress to date at 1 randomly selected prompt per day.

**Objective:**

The primary objectives are to (1) develop a real-time DD for giving participants personalized updates on their progress in the study and (2) examine its preliminary overall effects on study compliance and experiences. Secondary objectives are to identify person-, day-, and moment-level characteristics associated with study compliance and person-level characteristics associated with perceived usefulness of the DD.

**Methods:**

This is a protocol for Project ENGAGE, a 2-arm randomized controlled trial. Arm 1 (EMA group) is engaged in a standard EMA protocol, and arm 2 (EMA+DD group) is engaged in the same study but with additional personalization and feedback. Inclusion criteria are (1) previous participation in a recent college student survey about health behavior and mental health who indicated willingness to participate in future research studies and (2) indicated past-month alcohol use; lifetime marijuana, hashish, or Delta-8-tetrahydrocannabinol (THC) use; or some combination of these on that survey. All participants in this study completed a baseline survey; EMA at 11 AM, 2 PM, 5 PM, and 8 PM each day for 21 days; and an exit survey. Participants in arm 2 engaged in a microrandomized trial, receiving a personalized DD at 1 randomly selected prompt per day. Primary outcomes include whether a survey was completed, time to complete a survey, and subjective experiences in the study. Primary analyses will compare groups on overall study compliance and, for arm 2, use marginal models to assess the momentary effect of receiving 1 updated DD per day.

**Results:**

Approval was granted by the university’s institutional review board on February 8, 2023. Recruitment via direct email occurred on March 30 and April 6, 2023; data collection was completed by April 29, 2023. A total of 91 individuals participated in the study. Results have been accepted for publication in *JMIR Formative Research.*

**Conclusions:**

Results from the evaluation of this study will indicate whether providing (at randomly selected prompts) real-time, personalized feedback on a participant’s progress in an EMA study improves study compliance. Overall, this study will inform whether a simple, automated DD presenting study compliance and incentives earned to date may improve young adults’ compliance and engagement in intensive longitudinal studies.

**International Registered Report Identifier (IRRID):**

DERR1-10.2196/57664

## Introduction

### Overview

The prevalence of alcohol and cannabis use peaks in young adulthood (age range approximately 18-25 years), with 31% of young adults reporting recent heavy episodic drinking (5+ drinks in a row) and 11% reporting daily or near-daily cannabis use [1]. Heavy and frequent use in this age group can be linked with risk for alcohol use disorders, cannabis use disorders, or combined alcohol and cannabis use disorders, with 16.4% of young adults meeting criteria for a past-year alcohol use disorder and 16.5% meeting criteria for a past-year cannabis use disorder [2]. Far more common are the numerous acute substance-related harms that young adults experience from heavier alcohol use, cannabis use, or combined alcohol and cannabis use episodes in daily life, such as social-interpersonal issues, physical effects, and memory impairments [3].

Real-time data capture, as in daily diary studies or ecological momentary assessment (EMA) studies, is ideal and widely used for identifying antecedents and consequences of days and moments of highest alcohol- and cannabis-related risk [4]. Such intensive longitudinal data methods can be used to distinguish between- and within-person effects by asking questions such as “Are individuals who experience more stress more likely to report binge drinking?” (between-person) and “Are individuals more likely to report binge drinking on days when they experience more than their usual stress?” (within-person). Such methods are also ideal for capturing the dynamic nature of substance use behavior in daily life and identifying contextual risk factors that signal higher-risk episodes. Using such methods could ultimately provide a signal to trigger real-time intervention delivery.

However, compliance—particularly in substance use studies—can be a major concern for several reasons. First, young adults are less likely to complete reports on weekends when substance use is more likely to occur [5,6] and thus most critical to assess. Second, particular types of substance use behaviors, such as combining alcohol and cannabis, occurs somewhat rarely [7]. These behaviors can only be captured with many days of assessment, but participant compliance and motivation to participate in the study protocol may wane over time. Third, to assess behavior in optimal windows and provide timely intervention content, in some cases it is critical to assess behavior *in the moment when risk factors are higher*. However, engagement in the study protocol has the potential to interfere with participants’ daily lives. If young adults feel burdened by intensive longitudinal data collection, they may be less likely to provide complete data. A meta-analysis by Jones et al [8] concludes that overall, EMA compliance is only approximately 75% among individuals who use substances. Few studies have examined ways to improve participant compliance during intensive longitudinal data collection, yet recent work suggests that personalizing messages to young adults may increase compliance [9].

Mobile health apps have been developed in efforts to improve symptoms related to mental health, substance use, and a variety of other chronic health conditions and health behaviors. Many of these apps have shown no efficacy and/or modest clinical effects [10]. Poor engagement and related factors (eg, compliance, retention, and dropout) likely contribute to these findings because poor engagement with intervention content greatly weakens therapeutic effects. Only recently have studies of mobile health apps included measures of engagement as important outcomes; some even include aspects of engagement as the focus of interventions [11,12]. Among a wide variety of mobile health apps for managing chronic conditions (eg, mental well-being and HIV medication adherence), a meta-analysis estimated that the pooled dropout rate across apps was 43%, though the duration (range: 2 weeks to 1 year) and characteristics of study design varied considerably [13]. In another meta-analysis of apps addressing mental health problems, the dropout rate was approximately 36% after 8 weeks [11].

One way to improve young adult compliance may be to provide personalized, updated progress reports throughout their study participation, such as a data dashboard (DD) with real-time feedback on their progress in a study or aspects of the participant’s current or recent characteristics or behaviors. Personalization may be particularly useful because it can provide timely information unique to the person. Personalization may be operationalized in a variety of ways and may include content that serves self-interest or prosocial goals. For example, Carpenter et al [14] implemented a trial in which participants received biweekly messages about accrued reward points for a chance to win a prize for themselves (self-interest messages) or a chance to win a prize to donate to a charity (prosocial messages) and compared compliance rates. Interestingly, self-interest messages were more effective for compliance early in the study, and prosocial messages were more effective later in the study [14]. Thus, providing personalized data-driven feedback, particularly with the goal of improving compliance, is an understudied but particularly useful area of investigation.

To better understand study engagement and begin to address limitations in prior research, we enrolled young adults in a 2-arm randomized controlled trial called Project ENGAGE. Both groups participated in a 21-day EMA study that included 4 survey prompts per day (up to 84 prompts total). Participants were randomized to either participate in a traditional EMA study, where standard text prompts with links to a survey were received 4 times per day (EMA group), or an augmented study condition in which the text prompt was accompanied by a DD for 1 randomly selected survey per day throughout the EMA protocol (EMA+DD group). This 2-group design allows us to examine the overall effect of receiving 1 DD per day on overall study compliance and engagement. Individuals assigned to the EMA+DD group participated in a microrandomized trial (MRT) [15], which allows us to examine the short-term effects of the DD on momentary study compliance and inform the development of a just-in-time intervention that could be deployed to maximize study compliance within individuals.

### Aims and Objectives

#### Primary Objectives

The primary objectives of Project ENGAGE are to (1) develop a platform or delivery system for providing real-time, personalized updates on progress in the study and (2) examine the preliminary overall effects of embedding this real-time DD on study compliance and experiences. Intervention group differences will be examined in terms of the overall number of completed surveys (out of 84 prompts), average time to submit completed surveys, and subjective experiences with the study protocol. For the second aim, primary outcomes include participants’ overall compliance with the 84 surveys, mean time to complete a survey after receiving a prompt, and user experiences with the DD system.

#### Secondary Objectives

Secondary outcomes are to identify person-, day-, and moment-level characteristics associated with study compliance and person-level characteristics associated with perceived usefulness of the DD:

1. The following person-level characteristics will be examined in relation to study compliance overall and as moderated by the treatment group: baseline characteristics (gender, first and second vs third and fourth year in college, and history of substance use) and weekly app use.
2. We will estimate differences in compliance outcomes (did or did not complete all 4 surveys that day and mean time to complete surveys that day) as a function of day in study (days 1-21) and whether it was a weekday or weekend.
3. We will compare differences in momentary compliance and time to respond to a prompt across the following moment-level characteristics: whether the participant reported using their phone at the time they received the prompt, and (for the EMA+DD group) time of day on which the DD was received.

This study will yield didactic information for the broader research community on how to deploy a real-time DD or messaging system in a smartphone-based EMA study to improve ongoing participant compliance. Additionally, this study will demonstrate how to collect and analyze data from a study that uses an MRT embedded within a 2-arm randomized controlled trial.

## Methods

### Participants and Trial Design

Participants were drawn from a sample of undergraduate college students at a large public university who previously participated in a large, campus-wide cross-sectional survey in Spring 2023 and agreed to be contacted for future research. Participants who indicated past-month alcohol use or lifetime marijuana, hashish, or Δ-8-tetrahydrocannabinol (THC) use or some combination thereof were sent an email inviting them to participate in this study. Individuals interested in participating contacted the study team for more information or moved directly to the screener questionnaire using unique REDCap (Research Electronic Data Capture; Vanderbilt University) survey links provided in their emails.

Individuals were eligible to continue into the study if their responses to the screener questionnaire indicated that they were 18-25 years of age, used alcohol and cannabis in the past 30 days, and used an iPhone with an iOS version of 12 or more recent. The iPhone eligibility requirement was added to overcome technical challenges and additional confounding elements related to variations in how REDCap surveys and visual messages are displayed in iOS versus Android systems. Previous work by the study team has shown that students in the current institution are overwhelmingly iPhone users.

Eligible participants who consented to participate in this study were randomized into either the EMA group or the EMA+DD group and directed to the baseline survey. The randomization was performed automatically such that each participant had a 50% chance of being assigned to either of the 2 groups. The team planned to collect data from at least 40 individuals in each study condition. Study team members were able to see participants’ group assignment but had no contact with participants at any time to avoid introducing interview bias. After completing the baseline survey, participants were automatically enrolled in the 21-day portion of the study, with day 1 starting on the first Thursday after baseline completion. During the 21-day portion of the study, the participants received text-based prompts with a personalized REDCap link to a web survey.

Each prompt was sent at 4 equally spaced times per day (11 AM, 2 PM, 5 PM, and 8 PM) for 21 days, for a total of 84 prompts for each participant. Assessment timing was fixed to ensure data collection protocol as identical between both groups, to allow time in between assessments or avoid stacking of assessments, and to accommodate typical sleeping schedules and timing of substance use among our sample of college students who use substances*.* At each prompt, participants reported their substance use since the last prompt, psychosocial factors, and whether they were using their phone at the exact moment of receiving each prompt. Participants were instructed to complete each survey within 60 minutes of receiving the link, at which point the link would expire. At the conclusion of the 21-day portion of the study, participants completed an exit survey to provide feedback on their experiences in the study, including the acceptability and usefulness of a DD (for the EMA+DD group) or their openness to receiving a DD (for the EMA group).

### Real-Time Data Dashboard Intervention: an MRT Design

**Figure 1:**
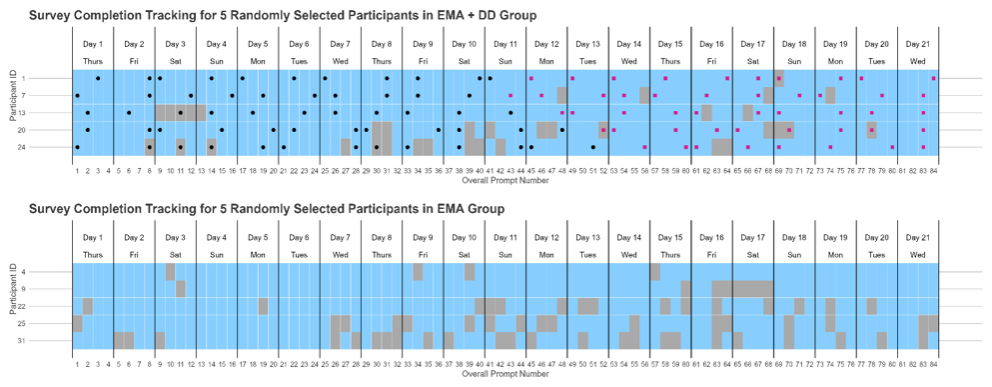
Data collection schedule and corresponding compliance data from 10 random study participants: 5 in the EMA+DD group (top panel) and 5 in the EMA group (bottom panel). Each row represents 1 participant, and each cell represents 1 day. Cell shading indicates EMA completion by the participant (blue=completed, gray=missing), and dots indicate display of the DD to the participant (black=DD without completion bonus progress ring, red=DD with completion bonus progress ring). DD: data dashboard; EMA: ecological momentary assessment.

For individuals in the EMA+DD group, 1 of the 4 prompts on each day included a DD component plus a personalized message, whereas the other 3 prompts simply presented the standard text with a link to the survey. Within each day, the prompt at which the DD would be delivered was selected at random. Figure 1 depicts the data collection schedule and corresponding compliance, as experienced by 5 random participants in each condition.

Our DD included several features to convey a participant’s study compliance. The visual image for the DD was partly inspired by the progress charts or exercise rings commonly found on wearable smart devices and fitness trackers (eg, Apple Watch and Garmin devices). Multiple iterations of the DD were tested internally by the study team to confirm accuracy and ensure that the information was user-friendly. The DD comprised 2 separate rings in the form of concentric circles. The outer ring (blue) represented the number of surveys that have been completed; the inner ring (red) represented the number of days (out of 21) completed. The colored parts (nongray areas) of the ring provided a visual representation of study compliance to date and were labeled with a count of the prompts and days completed to that point. Once participants completed at least 50% of their daily surveys, a portion of the outer ring turned yellow. The yellow portion indicated the number of surveys remaining before the participant reached a high completion bonus; the number of remaining surveys also was shown in the yellow portion of the ring. Color-matched text below the visual image provided a key to interpret the rings, with blue text showing the number of surveys completed, red text showing the number of days left in the study, and yellow text showing the number of surveys left before achieving the bonus (only when the bonus event activated, as described above). The center of the rings represented a “bank,” displaying the total amount of incentive money the participant had earned to that point in the study. The participant’s name also was generated on each DD as a form of personalization (eg, “[Name’s] progress so far!”). Colors for the rings were selected from a color palate that is more distinguishable for individuals with colorblindness.

**Figure 2:**
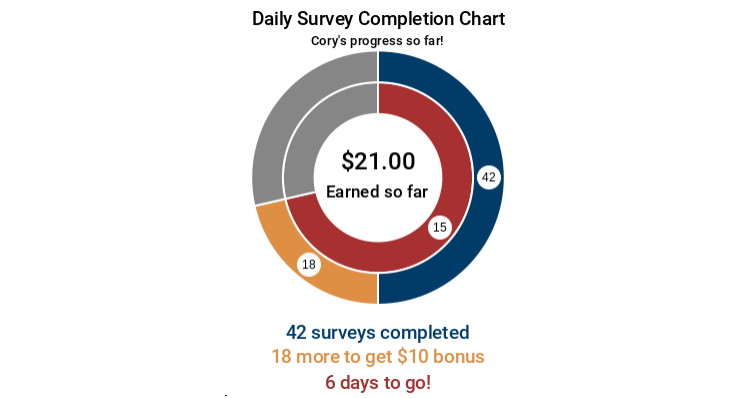
Example of a data dashboard that accompanied a text prompt to complete an ecological momentary assessment survey for a hypothetical participant who had completed 15 days of the study, provided data in response to 42 prompts to date, was 18 prompts away from reaching the high compliance bonus, and had earned US $21 in incentives.

Figure 2 shows a hypothetical example of the real-time, personalized DD that accompanied 1 randomly selected prompt per day among individuals randomized to the EMA+DD group. This example shows data for a hypothetical individual on day 15 who had completed 42 prompts, conveying the participant’s progress in the study to date. Specifically, the DD shows the number of prompts completed so far, the number of days remaining in the study, the number of surveys remaining until they earn the high completion bonus, and the total amount of incentives earned to date.

### Study Implementation

The entirety of the study was conducted in REDCap, which is a free survey platform that allows researchers to create and manage surveys to collect data securely. The complex study was designed and implemented in a way that was fully automated.

Specifically, we created a project consisting of multiple surveys: a screener, a baseline survey, 4 momentary surveys (1 for each time of day: 11 AM, 2 PM, 5 PM, and 8 PM) for the EMA portion of the study, and an exit survey. Surveys were used to store participants’ contact information separately from the rest of the data. These surveys were uploaded by the research team for all potential participants. This triggered the automated survey invitations for these individuals to go out at a scheduled date and time. At each prompt, these automated invitations provided unique participant links to the screener survey.

The screener survey contained the consent form, as well as several eligibility-related questions. Once a participant clicked on the link and completed the screener, they were told whether they were eligible to continue. The survey ended for participants who were not eligible to continue, but they were provided with study team contact information if they had further questions.

Participants who were eligible after the screener survey were automatically forwarded to the baseline survey. After completing the baseline survey, each participant was redirected to a server maintained by the institution’s survey research center. This server randomly assigned each participant to the EMA or the EMA+DD condition and scheduled all 84 prompt invitations via texts using Twilio’s Multimedia Messaging service. For the EMA group, all 84 invitations would simply include the text “It’s time to complete your next survey:” with a link to the EMA survey in REDCap corresponding to that time of day.

For the EMA+DD group, on each of the 21 study days, the server would randomly select 1 of 4 prompts at which to deliver the DD. At the other 3 prompts during that day, participants would receive the standard message that was used for all prompts in the EMA group. Just before each prompt, if a participant was randomly determined to receive a DD at that prompt, the server used JavaScript libraries to calculate the compliance data for each participant and then generated a visual image showing their progress to date in the study, to be delivered at that prompt.

Participants were given 1 hour to complete each survey after receiving the invitation via text. The invitation links in the text messages took the participant to the server and forwarded them to the relevant REDCap survey only if the link was clicked within the designated timeframe and the participant had not already completed that prompt.

At 11 AM on the day following the 21st day of the EMA study, all participants in both groups received a link to an exit survey, which assessed their experiences while participating in the study. Participants had 7 days to complete the exit survey.

### Outcome Measures

#### Primary Outcomes

This study focused on 2 objective measures of compliance and a set of quantitative and qualitative measures of subjective experiences. Primary outcomes included the following:

1. Overall compliance: overall compliance was derived from a moment-level binary measure of compliance (ie, an indicator of whether the participant completed the survey within 60 minutes of a particular prompt), summed across 84 prompts within an individual. This was used to address the question: “Did individuals who received a daily DD complete a greater number of surveys, on average, compared to those who did not?”
2. Average time of survey response: average time was derived from the length of time between the survey prompt and survey completion (among completed surveys), averaged across up to 84 prompts. This outcome was used to address the question: “Did participants who received the DD complete surveys more quickly, on average, compared to those who did not?”
3. Subjective experiences of the study protocol: subjective experiences were assessed in the exit survey and were compared across groups. This comparison relied on both quantitative and qualitative measures of experiences.

#### Secondary Outcomes

1. Total number of surveys completed within each week, to compare whether participants were more likely to comply to the EMA protocol during week 1, 2, or 3, and whether the intervention effect varied across weeks.
2. Mean time to complete surveys (among completed surveys) within each week.
3. Total number of surveys completed within each day, to compare whether participants were more likely to comply to the EMA protocol across days 1 through 21, and whether they were more likely to comply on weekdays versus weekends. These differences also were examined to see whether the intervention effect varied across days or types of days.
4. Mean time to complete surveys (among completed surveys) within each day.
5. The moment-level binary indicator of compliance and moment-level length of time to complete each survey will be used as outcomes in multilevel models examining moment-level predictors of compliance (eg, time of day and whether they completed last survey) and moment-level predictors of time to complete surveys (eg, whether currently using phone, stress, positive affect, and negative affect). Group assignment will be included as a moderator to see whether the intervention effect varies across types of moments.
6. (For the EMA+DD group only) The
  moment-level
  binary indicator of compliance and moment-level length of time to complete each survey will be examined in relation to whether the DD was included at that prompt.

### Ethical Considerations

The institutional review board at the Pennsylvania State University approved all study procedures (#00021945). Participants provided informed consent before completing the baseline and daily diaries. Study data are deidentified. Participants could earn up to US $67 via an e-gift card for full participation in the study. Specifically, participants were compensated US $10 for the baseline survey, US $0.50/daily survey, a US $10 bonus if they completed 70+ of the 84 surveys, and US $5 for completing a brief exit survey.

## Results

Recruitment invitations were sent via direct email to 411 individuals. Of these, 92 (22%) completed the consent form, completed the baseline survey, and were randomized to either the EMA group or the EMA+DD group. Among these individuals, 91 (99%) ultimately participated in the EMA survey (ie, provided data at 1 or more EMA prompt), thus determining our final sample size. Data were collected during March and April 2023.

Participants were predominantly White (92%), non-Hispanic (89%), and female (67%), with a mean age of 20.3 (SD 1.18) years. Among the 91 participants, 43 (47%) were randomized to the EMA group and 48 (53%) were randomized to the EMA+DD group. Overall study compliance was very good, with a mean of 64.3 (14.6) out of 84 (76.5%) prompts completed, resulting in a total of 5896 (out of a possible 7644) person-moments of data.

## Discussion

Results from our evaluation of the MRT will provide evidence about whether providing real-time and personalized feedback on a participant’s progress in an EMA study improves compliance across multiple metrics. MRTs have a high potential for informing the development of effective just-in-time interventions focused on higher-risk substance use behavior. Information about the use of personalized dashboards to improve compliance will be a critical finding from this study, as compliance is a critical first step toward greater overall study engagement. Evaluating the effect of receiving a DD on speed of completing that particular survey will also be useful, as it reflects a state of investment or energy and cognitive energies, key components of engagement [16].

Further, REDCap and similar data collection tools are ubiquitous in most university settings. Thus, our automated approach could be deployed at many institutions with existing technology. This approach circumvents the often tedious or slow process of gaining approval for use of a third-party service to deploy such studies. Although REDCap does not natively support EMA data collection, there are relatively straightforward workarounds to automate a tailored process such as in this study. Because of the nearly universal adoption of these software packages, a wealth of information including community forums also exists online to troubleshoot issues and develop new techniques.

This study necessitated a specialized system to implement the generation of the DD based on real-time information of the participant. Through a collaboration with the institution’s survey research center, expert staff were able to connect the REDCap system to a server in which custom code was used to determine the timing of deployment, calculate data for inclusion, and generate the DD for each individual at appropriate times.

This feasibility study was limited in sample size and thus may be underpowered to evaluate the effectiveness of the DD. Further, generalizability to the broader population of young adults may be limited given that all participants were currently attending college at a large, predominantly White, public university in the Northeast region of the United States. Additionally, the system designed for this study was specific for iPhone users; additional modifications would be necessary to adapt the study for Android phone users.

Overall, this study will inform whether a simple, automated DD that presents study compliance and incentives earned to date could improve ongoing compliance and overall engagement in intensive longitudinal studies. Future research could expand this scope by varying the frequency of presenting a DD (including providing one at every assessment point) and adding additional information to the graphical presentation of data. Additionally, real-time data such as biofeedback data from wearable devices could be integrated in future studies to further personalize messaging. For example, a heart rate monitor could be used to tailor messaging that corresponds to a DD, such as “Your heart rate was really high this afternoon. You said you like to meditate—might be a good idea to try it now.” Such personalization could help researchers to better understand and improve on the effects of providing a personalized DD and use it to trigger tailored intervention content. Alternatively, future studies might take into consideration relevant, passively detected contextual features such as current weather to determine whether they moderate the effect of receiving a DD.

## Abbreviations

DD: data dashboard
EMA: ecological momentary assessment
MRT: microrandomized trial
REDCap: Research Electronic Data Capture
THC: Δ-8-tetrahydrocannabinol

## Data Availability

Data from this study are available from the corresponding author on reasonable request.
